# Supplementary figures and images for: Establishment of a Replicon Reporter of the Emerging Tick-Borne Bourbon Virus and Use It for Evaluation of Antivirals
Source: Front Microbiol. 2020 Sep 8;11:572631. doi: 10.3389/fmicb.2020.572631 (PMC7506111; doi:10.3389/fmicb.2020.572631)

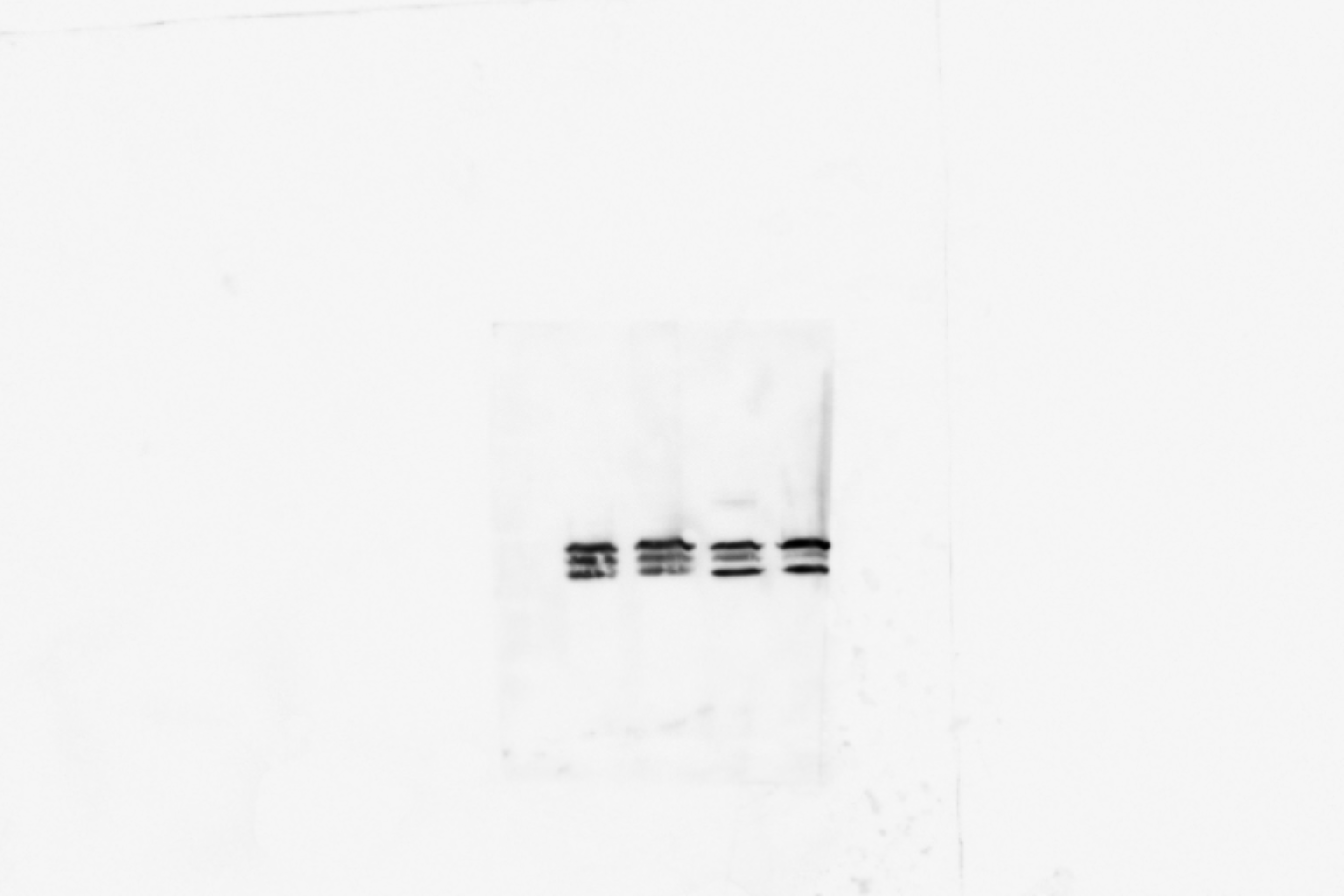

Supplement: Supplementary file 1 [file Data_Sheet_1.ZIP › Original images of Figure 2/Actin.tif]

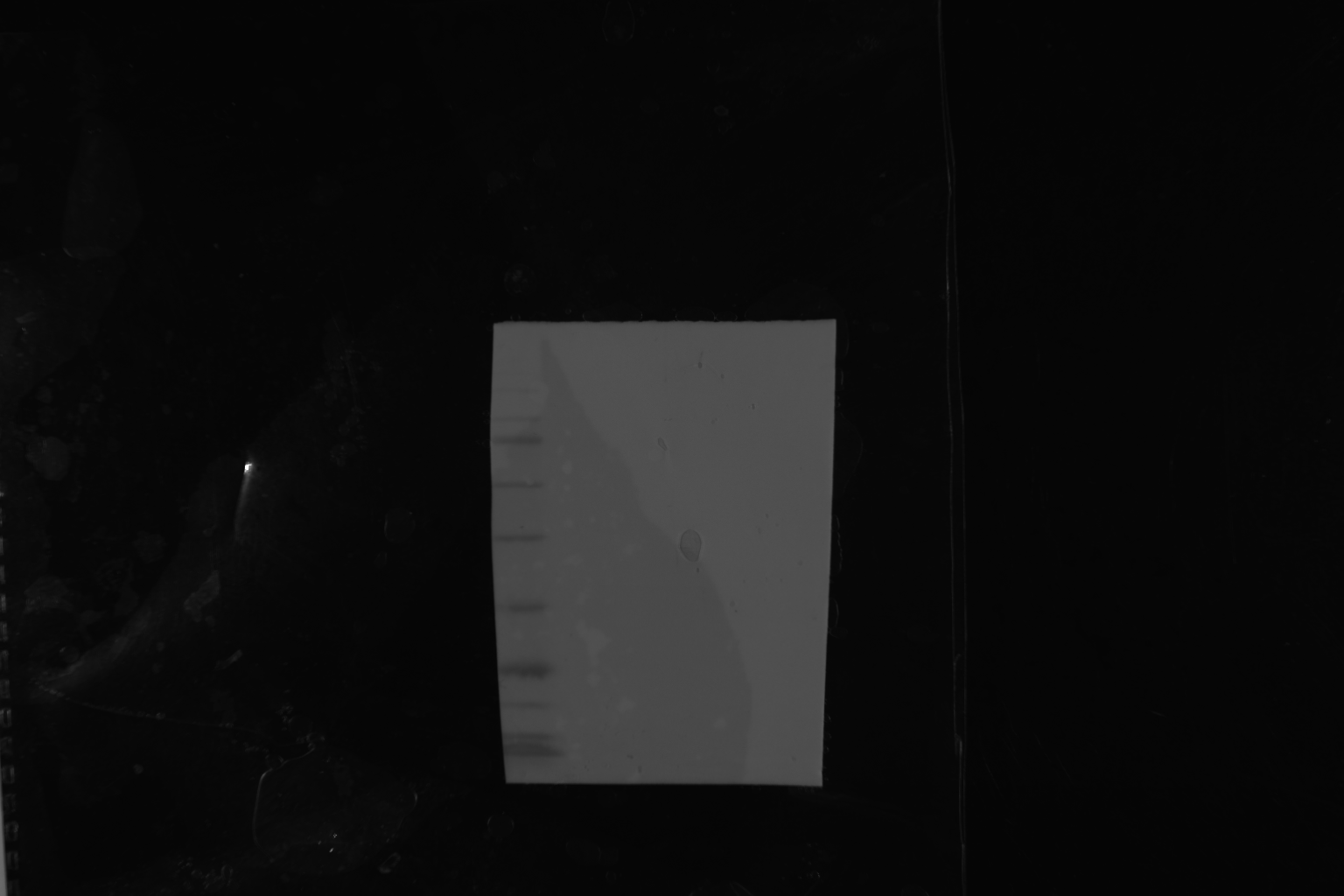

Supplement: Supplementary file 1 [file Data_Sheet_1.ZIP › Original images of Figure 2/Actin_marker.tif]

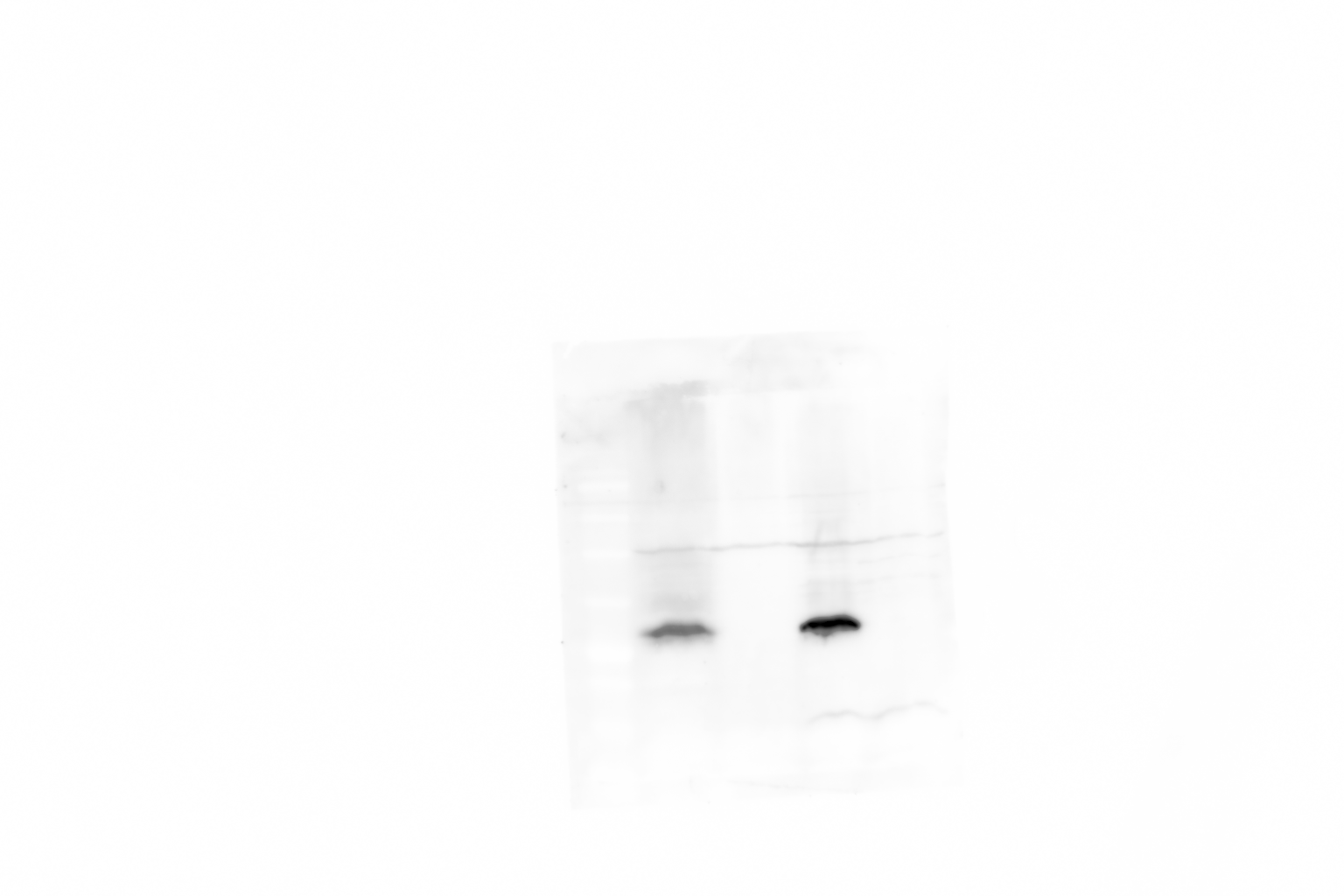

Supplement: Supplementary file 1 [file Data_Sheet_1.ZIP › Original images of Figure 2/M protein.tif]

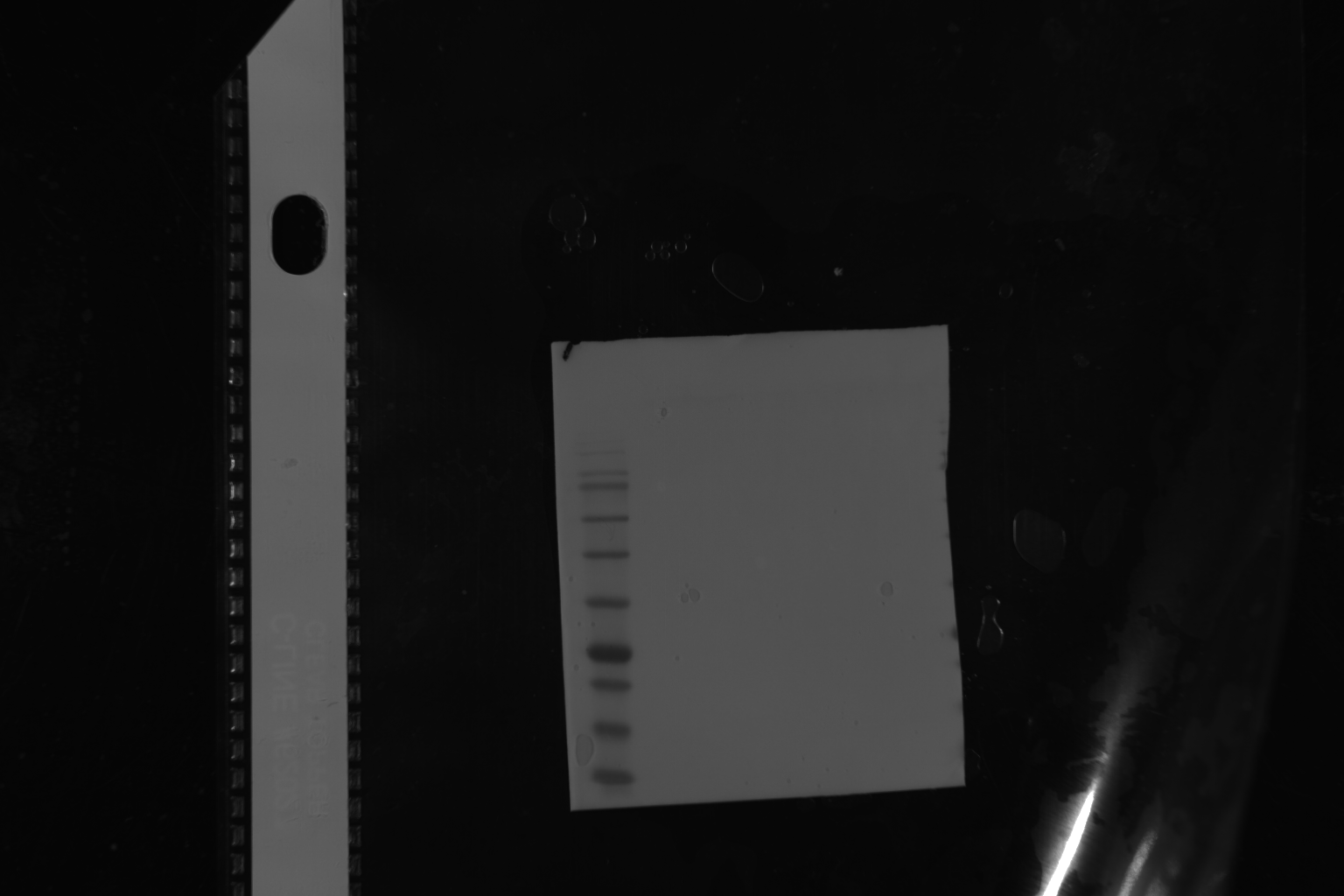

Supplement: Supplementary file 1 [file Data_Sheet_1.ZIP › Original images of Figure 2/M-marker.tif]
